# CRISPR-Cas9 delivery strategies with engineered extracellular vesicles

**DOI:** 10.1016/j.omtn.2023.102040

**Published:** 2023-09-26

**Authors:** Yaoyao Lu, Kelly Godbout, Gabriel Lamothe, Jacques P. Tremblay

**Affiliations:** 1 Centre de Recherche du CHU de Québec -Université Laval, Québec city, QC G1V4G2, Canada

**Keywords:** MT: Exploiting Extracellular Vesicles as Therapeutic Agents Special Issue, extracellular vesicles, CRISPR-Cas9, gene therapy, delivery, tissue targeting

## Abstract

Therapeutic genome editing has the potential to cure diseases by directly correcting genetic mutations in tissues and cells. Recent progress in the CRISPR-Cas9 systems has led to breakthroughs in gene editing tools because of its high orthogonality, versatility, and efficiency. However, its safe and effective administration to target organs in patients is a major hurdle. Extracellular vesicles (EVs) are endogenous membranous particles secreted spontaneously by all cells. They are key actors in cell-to-cell communication, allowing the exchange of select molecules such as proteins, lipids, and RNAs to induce functional changes in the recipient cells. Recently, EVs have displayed their potential for trafficking the CRISPR-Cas9 system during or after their formation. In this review, we highlight recent developments in EV loading, surface functionalization, and strategies for increasing the efficiency of delivering CRISPR-Cas9 to tissues, organs, and cells for eventual use in gene therapies.

## Graphical abstract

**Figure:**
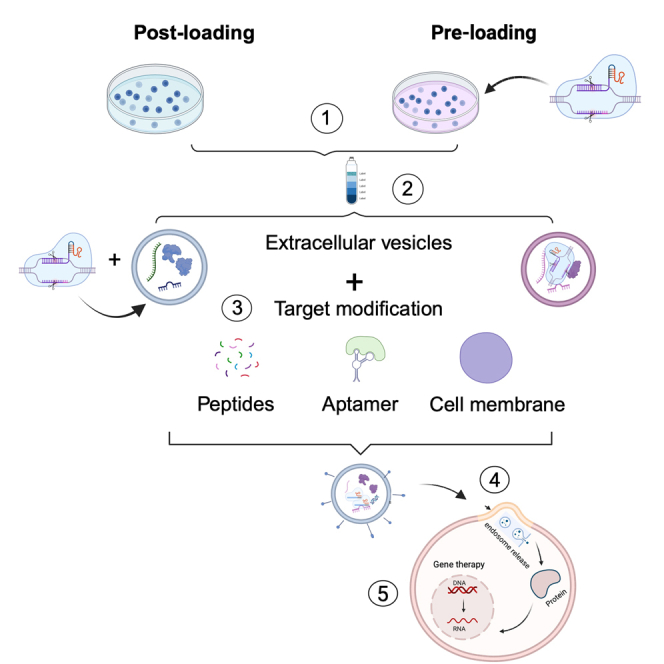

## Teaser

Lu and colleagues highlight recent developments in engineered EVs for CRISPR-Cas9 delivery in the context of gene therapy. We explain the main methods to load this system into EVs. We also present different strategies for EV surface functionalization to promote target delivery to specific organs.

## Introduction

Genetic engineering is a sophisticated science that modifies genes at the molecular level. External genes can be replicated, transcribed, and expressed in receptor cells to produce a variety of valuable proteins. This expression can then achieve a functional modification of the receptor cells. The discovery of CRISPR-Cas systems has ushered in a new era of genome editing. The underlying CRISPR-Cas gene editing technology comes from the bacteria’s defense system against phages. There it evolved to cut invading nucleic acids and confer resistance to phages that previously infected the cell. In practice, the CRISPR-Cas protein needs to combine with a single artificial synthetic guide RNA (sgRNA) generated by fusing the CRISPR RNA (crRNA) and trans-activating crRNA. This forms the Cas and sgRNA ribonucleoprotein (RNP), which targets and cleaves specific DNA,^1^^,^^2^ which is programming of CRISPR-based gene editing tools. Type II CRISPR-Cas9 is now widely used in multiple fields requiring gene editing, as it is simple to use and highly efficient.^1^^,^^2^ To date, it has been used in basic biology, healthcare, and agriculture research. However, the widespread use of this technology in therapies is hindered by its accidental activity on non-target gene effects when editing genes in cells, the human autoimmune response against Cas9, and the danger associated with inefficient delivery approaches. To implement the use of CRISPR-Cas9 in gene therapies, developing a safe and efficient *in vivo* delivery system remains the most challenging aspect.^3^^,^^4^

Extracellular vesicles (EVs) are endogenous membranous particles secreted spontaneously by all cells. They function as messengers by exchanging cargo between cells, allowing the transport of various signaling chemicals, such as bioactive lipids, proteins, and nucleic acids (DNA and RNA).^5^ There are three primary types of EVs classified based on their size, function, and biogenesis pathway: exosomes, ectosomes, and apoptotic bodies.^6^ Exosomes (30–150 nm) are formed as intraluminal vesicles through the inward budding of early endosomes and are subsequently released into the extracellular space. Ectosomes are formed by the outward budding or pinching of the plasma membrane of cells. They contain different types of vesicles, which range from less than 100 nm to several micrometers. Ectosomes are mainly divided into microvesicles (0.2–1 μm) and large oncosomes (>1 μm). Apoptotic bodies (1–5 μm) contain harmful cellular components left over from apoptosis.^7^ Additional EV subtypes, such as migrasomes, have recently been described and are still being studied.^6^^,^^8^ The evidence accumulating reveals significant differences in the characteristics, composition, and function between these distinct EV types. However, they are involved in pathological and physiological states by transmitting bioactive components and overcoming biological barriers.^9^^,^^10^ Based on these characteristics, EVs are increasingly being used to deliver drugs. The EVs mentioned below represent exosomes and microvesicles.

In this review, we discuss current developments in the delivery of CRISPR-Cas9 using EVs while also outlining strategies to increase the effectiveness of EV delivery approaches.

### CRISPR-Cas9 delivery approaches

The RNP complex has difficulty passing the cell membrane because Cas9 is a large protein (160 kDa), and the sgRNA is negatively charged. To date, many strategies have been developed to deliver CRISPR-Cas9 to target cells. These can be categorized as viral vectors (adenovirus, adeno-associated viruses ([AVs] and lentivirus) and non-viral vector-based delivery strategies. Non-viral vector approaches can be further subdivided into natural/synthetic materials (e.g., EVs, penetrating peptides, lipid nanoparticles [LNPs], and polymer nanoparticles) and physical approaches (e.g., cell microinjection, electroporation, sonoporation, and laser irradiation).^11^ To date, viral vectors have demonstrated the best CRISPR-Cas9 delivery and gene editing efficiency. AAVs, as the most widely used viral vector, display unique advantages such as reduced immunogenicity and mild toxicity compared with other viruses.^12^^,^^13^^,^^14^ However, the packaging capacity of AVVs (<5.2 kb) is a limiting factor when delivering the components of CRISPR-Cas9 and its derived gene editing technologies.^15^^,^^16^ Indeed, the open reading frame encoding *Streptococcus pyogenes* Cas9 is approximately 4.2 kb.^4^ Furthermore, the CRISPR-Cas9-derived gene editing technologies, including base editors (4.2–5.2 kb)^17^ and the prime editor (～6.3 kb)^18^ are larger than the capacity of AVVs. Moreover, this issue is compounded when sgRNAs and prime editing guide RNAs must be delivered in parallel.^19^ Although adenoviruses and lentiviruses have a greater capacity than AAVs, they demonstrate their own limitations and adverse effects. Adenoviruses induce an intense immune response, and lentiviruses are known to integrate into the host’s genome.

To increase the efficacy, safety, and targeting of gene editing, the method of delivering the CRISPR-Cas9 system must be actively considered. New strategies such as LNPs, EVs, and virus-like particles have been developed to deliver gene therapies.^20^ The clinical translation of these vectors have two primary challenges: safety and quick clearance by the reticuloendothelial system or the mononuclear phagocyte system. Cell-derived nanovesicles outperform manufactured nanocarriers in both aspects. EVs can also encapsulate dsDNA ranging from 100 bp to 20 kb with low immunogenicity.^21^^,^^22^ Additionally, the proinflammatory cytokines induced by EVs are both smaller in quantity and in effect when compared with inflammatory responses reported with LNPs.^23^^,^^24^ When considering the above and that the EV membrane structure can shield proteins and nucleic acids from the immune system and serum endonucleases,^25^ EVs are an excellent candidate for CRISPR-Cas9 delivery.

## The advantages and limitations of EV delivery

When delivering therapeutic cargo, EVs provide several advantages including a larger capacity, higher biocompatibility, lower host immune response, and immunogenicity compared with their leading competitors.^26^^,^^27^ Furthermore, their naturally occurring lipid and surface protein composition allows them to avoid phagocytosis, increases their half-life in blood, and reduces long-term safety concerns after their administration. The unique structure of EVs also protects their contents from destruction in the extracellular environment for prolonged periods.^7^^,^^28^ Additionally, the small size of EVs makes it easier for them to extravasate, move across physical barriers, and pass through the extracellular matrix. Studies on the impact of EVs and their cargo have been conducted on numerous disorders, including cardiovascular disease,^29^ type 2 diabetes mellitus,^30^ and cancer.^31^ Meanwhile, EVs were reportedly loaded with therapeutic agents, such as small interfering RNA (siRNA),^32^ microRNA,^33^ mRNA/proteins,^34^^,^^35^ CRISPR-Cas9,^25^ and drugs^36^ for treating disorders *in vitro* and *in vivo*. Phase I and II clinical trials based on EVs are being carried out to evaluate the efficiency of using bone marrow mesenchymal stem cell (MSC)-derived exosomes to deliver drugs in patients hospitalized with severe acute respiratory syndrome coronavirus 2 pneumonia and COVID-19 within 28 days of the first treatment (NCT04602442 and NCT04276987). The result showed that, in seven COVID-19 patients, MSC-derived exosomes were feasible to deliver drugs in clinic and well tolerated, with no evidence of prespecified adverse events, acute clinical instability, or dose-relevant toxicity at any of the doses examined.^37^

Conversely, various obstacles must be cleared before EV-based therapies may be implemented in clinics. First, many characteristics and mechanisms relating to EV biology remain elusive, such as how to increase the purity of the EVs by distinguishing the overlap size among EVs categories or other substances like lipoprotein, pili, tamm-Horsfall protein, and cell dribs.^38^ Second, the mechanism of production or absorption is still poorly understood. EVs from the same cell types may have paradoxical effects because of the variation in cell culture conditions and differences in purification techniques.^39^ Moreover, no universal sample collection protocol exists and there are no universal biomarkers for validation.^40^ Finally, solutions for the difficulties faced during cargo-loading, EV uptake, cargo release, and endosomal escape cell/tissue targeting efficiency are all still in their infancy. These factors need to be further investigated before the widespread adoption of EVs-based therapies.

### Methods to load cargo into EVs

Loading cargo into EVs is classified into endogenous and exogenous post-isolation loading methods.^41^ As the name implies, endogenous methods involve loading the cargo before the isolation of the EVs. This is accomplished by adding new genetic material or selecting molecules to interact with or modify parent cells, such as drugs, RNAs, DNAs, proteins, or select molecules that can be incorporated into their EVs.^42^ Exogenous post-isolation loading approaches encompass various techniques to load therapeutic cargo into EVs after their isolation.^43^^,^^44^ Each approach has its advantages and limitations.

During the endogenous loading method, EVs maintain their form and characteristics regardless of their cargo’s physical and chemical properties. However, this method has a relatively low loading efficiency, which limits its application as a standalone method. It is, therefore, effective when delivering small molecular proteins into EVs but ineffective when delivering biomacromolecules (proteins and nucleic acids) such as Cas9 protein or the dystrophin gene. Furthermore, proteins of interest can be incorporated in both the extra-vesicular and the intra-vesicular sides simultaneously, which could lead to immune responses against the EVs or the denaturation of external proteins during transport.

Compared with endogenous loading methods, exogenous post-isolation techniques allow a broader array of therapeutics to be loaded without altering the parental cell line. Since the cell types are not restricted to those that are easily transfected or have intrinsic cell regulation that allows chemicals to be inserted into cells, exogenous post-isolation methods are amenable to a wider range of EV-producer cells and can additionally be standardized and controlled. This is in contrast with endogenous loading, which depends on EV biogenesis and cell states.^45^

Whether one is using endogenous or exogenous loading techniques, the cargo is loaded either passively or actively. Passive approaches involve attracting bioactive substances into EVs by simple incubation. This is appropriate for small compounds like chemotherapeutic drugs and small nucleic acids. Hydrophobic chemicals have the best loading efficacy because they can bind to the cell membrane and the EV lipid bilayer in a stable fashion. However, the cargo enrichment in EVs by the passive loading approach depends on the condition of source cells and demonstrates poor repeatability.

**Figure 1:**
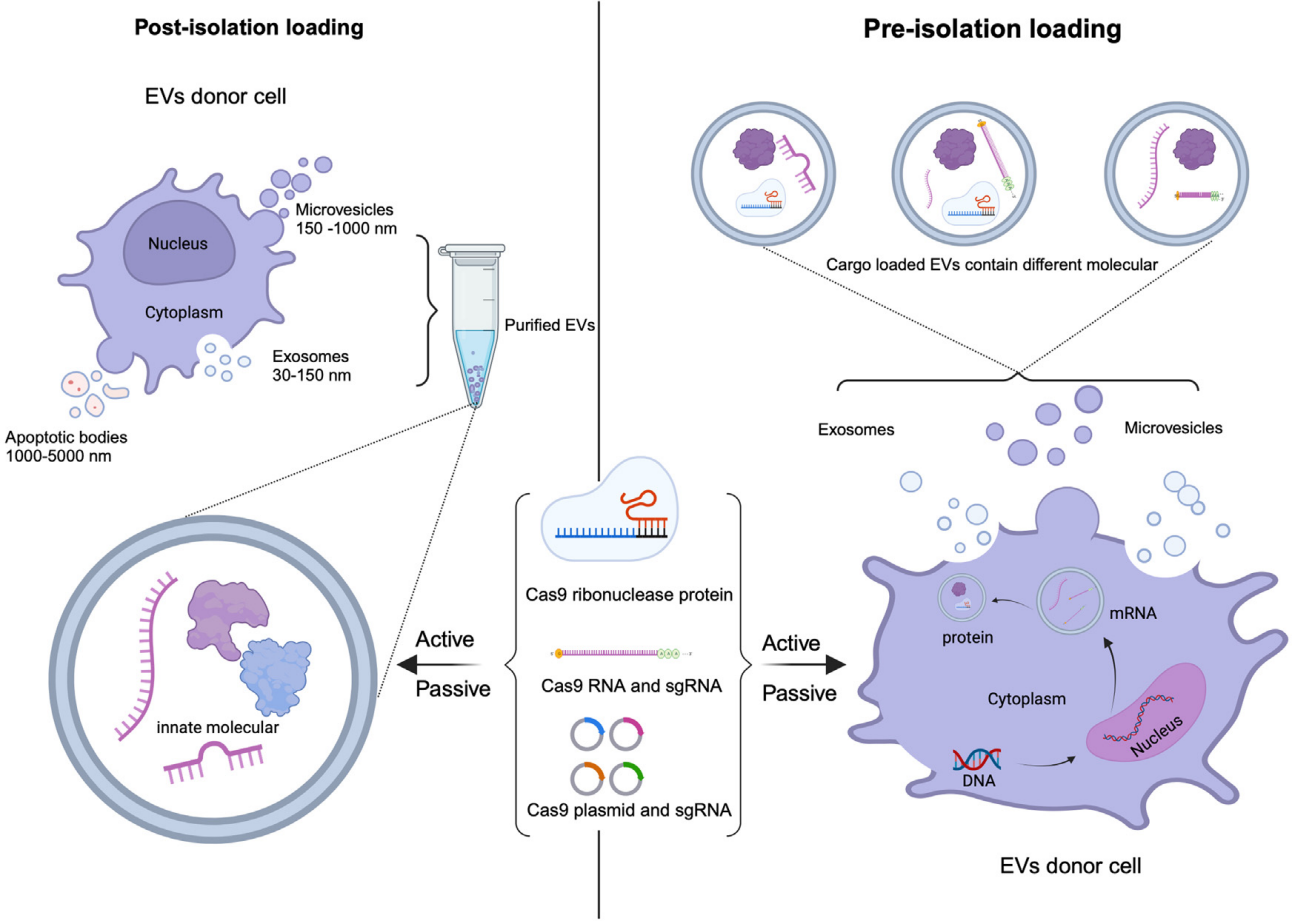
Methods for loading CRISPR-Cas9 components into EVs (Left) Post-isolation loading method. EVs are produced by unmodified parent cells. The components of the CRISPR-Cas9 system are loaded into EVs after their isolation and purification. (Right) Pre-isolation loading method. The components of the CRISPR-Cas9 system are transfected into parent cells. EVs are harvested and will contain the exogenous nucleic acid material by natural EV production route.

Active loading involves loading cargo into EVs by sonication, electroporation, freeze-thaw cycles, extrusion, and transfection (extensively reviewed elsewhere^46^^,^^47^). These methods generate higher loading efficiency than passive loading. However, these approaches share a common drawback in that the membrane of EVs can be damaged, affecting their structure and potentially affecting their ability to transport their materials. Based on those loading methods, the methods for loading the components of CRISPR-Cas9 into EVs are shown in Figure 1.

### Delivery of CRISPR-Cas9 using EVs

The CRISPR-Cas9 system can be used in mammalian cell cultures by incorporating it into the cells in the form of plasmids, mRNA, and protein. Plasmids are the most complex form, but are inexpensive to produce and simple to use. Once the Cas9 plasmid is delivered into the cytoplasm of target cells, it is sent to the nucleus and transcribed into mRNAs. Subsequently, the Cas9 mRNAs are transported to the cytoplasm and translated into Cas9 proteins. Part of the plasmid also encodes a sgRNA that later interacts with the Cas9 protein to form the Cas9/sgRNA RNP complex. Given the complexity of transfection, expression of Cas9 and its sgRNA, and gene editing, many techniques have been developed to deliver the Cas9/sgRNA complex at different stages of its development. Here, we further describe the details of delivering the different formats of CRISPR-Cas9 components *in vivo* and *in vitro* by EVs.

### EV delivering CRISPR-Cas9 plasmid

Kim et al.^48^ electroporated the CRISPR-Cas9 plasmid and sgRNA into SKOV3 cancer cell-derived exosomes to target the poly (ADP-ribose) polymerase-1 (*PARP-1*) gene in SKOV3 tumor cells and SKOV3 xenograft mice. The authors showed that their treatment-induced 55% insertions or deletions (indels) in *PARP-1*-positive SKOV3 cells and induced SKOV3 cell apoptosis in mice with ovarian cancer. They also demonstrated that cancer cells preferentially take up cancer cell-derived exosomes compared with exosomes produced by epithelial cells. This result shows that specific tropism can influence the ability of EVs to target specific cell types.

Furthermore, McAndrews et al.^49^ used a transfection reagent (Exo-Fect) to transfect the Cas9/sgRNA plasmid into exosomes derived from human embryonic kidney (HEK293T) epithelial cells. This allowed them to target the *Kras*^*G12D*^ oncogenic allele in pancreatic cancer cells and led to a 58% knockdown of the target *Kras*^*G12D*^ transcript levels *in vitro*. The authors also observed a decrease in the proliferation of tumor cells *in vitro* and of tumor growth inhibition *in vivo*. In a similar vein, exosomes are currently being investigated in phase 1 clinical trials in patients with metastatic pancreatic cancer with the *Kras*^*G12D*^ gene mutation by delivering *Kras*^*G12D*^ siRNA (NCT03608631).

Cell uptake efficiency is another consideration for exosome-mediated gene modification. Lin et al.^50^ described a simple incubation-based exosome-liposome hybrid system. They incubated exosomes with a mixture of liposomes and Cas9/sgRNA plasmids for 12 h at 37°C. They found that an incubation time of 12 h was best for balancing the fusion efficiency and stability. This strategy gave a higher loading capacity and a more effective delivery than native exosomes for delivering CRISPR-Cas9 to MSCs. Based on their outcomes, Liang et al.^51^ constructed chondrocyte affinity peptide-targeting exosomes (CAP-Exo). Those EVs are made by genetically adding a CAP at the N-terminus of the exosome surface protein Lamp2b. They transfected the CAP-FLAG-Lamp2b plasmid with Lipofectamine 2000 into dendritic cells to produce the CAP-Exo. The CAP peptide present on the exosome surface enables the exosomes to target chondrocytes and keep them in the joint following intra-articular injection. CAP-Exo was incubated with liposomes to encapsulate the CRISPR-Cas9 plasmid to knock down the matrix metalloproteinase 13 (*MMP-13*) in chondrocytes. The hybrid CAP-Exo successfully suppressed the production of *MMP-13* in chondrocytes, penetrated the deep region of the cartilage matrix in osteoarthritis rats, and attenuated the hydrolytic breakdown of the extracellular matrix proteins in the cartilage.

To target spinal cord injuries (SCI), Wang et al.^52^ modified human umbilical cord MSC-derived exosomes to display the CAQK peptide (a sequence that can specifically bind to the injured site after SCI). This system achieved tissue recovery by inhibiting the tumor necrosis factor α (TNF-α)-induced inflammatory response and restraining the activation of the TNF-α/nuclear factor κB signaling pathway.

Moreover, Xu et al.^53^ transduced the anti-CD19 chimeric antigen receptor (CAR) lentivirus into the HEK293T cells. EVs derived from anti-CD19-CAR-HEK293T cells express anti-CD19-CAR on the surface showed selective tropism for tumors. The outcome demonstrated that CAR-EVs accumulated in cancer tumors more rapidly than ordinary EVs. Their results also showed that the CRISPR-Cas9 system was effectively released both *in vitro* and *in vivo*, and precisely targeted the *MYC* oncogene.

We have compiled the research on post-isolation loading of CRISPR-Cas9 plasmids into EVs in Table 1. More information on how to modify the plasmids for delivering this cargo into EVs is provided elsewhere.^54^^,^^55^^,^^56^^,^^57^^,^^58^

**Table 1:** Delivery of CRISPR-Cas9 plasmid by EVs

EV source	Target organ/cells	Target gene	Loading method	Reference
SKOV3/HEK293T	tumor generated by SKOV3 xenografts	*PARP-1*	electroporation	Kim et al.^48^
HEK293FT	MSCs	*hCTNNB1*	liposomes	Lin et al.^50^
HEK293T	tumor generated by Raji-bearing xenograft	*MYC*	electroporation	Xu et al.^53^
HEK293T	pancreatic cancer	*KrasG12D*	Exo-Fect	McAndrews et al.^49^
DCs	chondrocytes	*MMP-13*	Lipofectamine 2000	Liang et al.^51^
MSC	spinal cord	*TNF-α*	electroporation	Wang et al.^52^

### EV delivering CRISPR-Cas9 mRNA

Delivering Cas9 to cells as an mRNA minimizes immunogenicity and carcinogenicity. In this form, Cas9 mRNA does not need to go to the nucleus or go through the transcription step. Translation of the mRNA can take place directly in the cytoplasm. However, when compared with delivering plasmids, mRNA is unstable. Its short half-life may decrease the effectiveness of the gene editing treatment. Furthermore, numerous studies have demonstrated that the bulk of mRNA in EVs are degraded to some extent. Besides, the EVs from different cell sources primarily include short (100 nt) RNA molecules and only trace amounts of full-length mRNA.^59^^,^^60^^,^^61^ Notably, exosomes exhibit poor encapsulation effectiveness when delivering bulky CRISPR-Cas9 mRNA, even when using efficient electroporation loading methods.^61^ Wei et al.^62^ reported that exosomes from glioma cells only contain endogenous mRNA copies varying from 1/10 to 1/100,000 exosomes are detected.

Despite the difficulties, CRISPR-Cas9 mRNA has been delivered to target cells by loading it into EVs via electroporation. One study^63^ demonstrated that EVs derived from red blood cells (RBCEVs) enabled editing in leukemia and breast cancer cells *in vitro* and *in vivo*. The author electroporated HA-tag Cas9 mRNA into RBCEVs to treat MOLM13 leukemia cells. After 48 h of incubation, the Cas9 protein was detected using immunostaining in 50% of MOLM13 cell nuclei. This study demonstrated an effective RBCEV treatment without generating any observable cytotoxicity. In addition, RBCEVs do not pose a risk for horizontal gene transfer; RBCs are enucleated cells lacking their own DNA.

To insert Cas9 mRNA into exosomes, Li et al.^64^ used surface-functionalized exosomes. Their EVs were decorated with a fusion protein, consisting of the exosome membrane protein CD9 fused to the RNA binding protein HuR. Through the interaction of HuR and AU-rich elements of the RNA, CD9-HuR exosomes efficiently loaded functional CRISPR-dCas9 mRNA into exosomes. The result showed that CRISPR-dCas9 dramatically decreased C/ebpα endogenous expression in the presence of C/ebpα gRNA.

More recently, de Jong et al.^65^ developed a CROSS-FIRE reporter system to validate the transfer of RNA into cells that already express the Cas9 protein. With this system, they demonstrated that the deletion of the exosome-related genes Alix and Rab27A, which are involved in exosome release and production, respectively, drastically decreased reporter cell activation.^65^

The studies to load Cas9 mRNA into EVs have been summarized in Table 2. In addition, other modifications shown in reference that succeeded in delivering mRNA into EVs could be considered when attempting to deliver Cas9 mRNA.^66^^,^^67^^,^^68^^,^^69^^,^^70^^,^^71^^,^^72^

**Table 2:** Delivery of CRISPR-Cas9 mRNA by EVs

EV source	Target organ/cells	Target gene	Loading method	Reference
Red blood cells	MOLM13 cells	miR-125b	electroporation	Usman et al.^63^
MDA-MB-231 cells	HEK293T	Reporter cell (mCherry)	transfection (Lipofectamine2000)	de Jong et al.^65^
HEK293T cells	Liver	C/ebpα	transfection (Lipofectamine2000)	Li et al.^64^

### Delivering CRISPR-Cas9 RNPs using EVs

The most efficient approach to deliver Cas9 for use in gene therapies involves trafficking the RNP directly. This circumvents the process of transcription and translation in target cells.^73^ Moreover, fewer off-target effects are detected when delivering RNPs directly.^74^ However, Cas9 is a large protein that hinders its loading into EVs. Current approaches to load RNPs into EVs can be broadly divided into four categories: (i) creating a fusion protein by linking the Cas9 protein to transmembrane or luminal proteins that are expressed specifically in EVs; (ii) performing post-translational modifications (PTMs) of the Cas9 protein such as ubiquitylation, phosphorylation, and glycosylation to load them into EVs; (iii) assisting by virus-derived protein like vesicular stomatitis virus glycoprotein (VSVG), help Cas9 load onto exosomes and increase the protein deliver efficiency via a pseudotyping mechanism; and (iv) incubating the Cas9 protein with EVs either with or without a transfection reagent to favor their incorporation.

Several teams have succeeded in loading the Cas9 protein into EVs by fusing it to a protein involved in EV proteins. For the protein mediating the surface and luminal engineering, Yao et al.^75^ use RNA aptamer Com sequence integrated into sgRNA loop and subsequently fused aptamer-binding protein Com into both terminals of CD63. The Com/com interaction enriched CRISPR-Cas9 and adenine base editor RNPs into EVs. They demonstrated that those EVs generated a 0.2% indel rate after being injected into the tibialis anterior muscle of Duchenne muscular dystrophy X-linked (DMD/mdx) mouse by intramuscular injection. Similarly, Osteikoetxea et al.^76^ reported that reversible heterodimerization of Cas9-fusions with EV sorting partners (cryptochrome 2 in combination with CD9 or a myristylation-palmitoylation-palmitoylation lipid modification) led to Cas9 being encapsulated into EVs. This approach efficiently loaded approximately 25 Cas9 molecules per EV and knocked down the PCSK9 gene with 6% indel efficiency in HEK293T cells.

Many methods have been developed to load Cas9 into EVs using PTMs. Whitley et al.^77^ fused an octapeptide derived from the leading sequence of the N-terminus of Src kinase to the N-terminal of Cas9. This led to the myristylation of the Cas9 and its subsequent packaging into EVs. This generated an eGFP knock-out efficiency of up to 42% when using VSVG-coated EVs. Wang et al.^78^ reported that they could pack ubiquitinated proteins into ARRDC1-mediated micro-vesicles (ARMM). To apply this to Cas9, they fused the WW domain from Nedd4 family members (ITCH, Nedd4-L, wwp2, and wwp1) to the protein, triggering its ubiquitination. The WW domain then interacted with the PPXY motif of ARRDC1 to load the Cas9 into ARMMs. This approach restored 12% of GFP expression in U2OS GFP-negative cells. In recent years, many other PTMs have been shown to mediate the entry of proteins into EVs. However, those modifications have not yet been used to deliver Cas9 into EVs.^79^^,^^80^

Virus proteins can also be used to facilitate the loading of the Cas9 into EVs while simultaneously increasing EV uptake by target cells.^81^^,^^82^ For example, Meyer et al.^81^ studied the impact of VSVG expression in cells internalizing EV. The VSVG protein is incorporated into the viral envelope when the membrane rafts are budding at the plasma membrane.^83^ When VSVG was expressed, the loading of the cargo protein was enhanced, and EV uptake was increased. Similarly, Gee et al.^84^ developed a new approach called "NanoMEDIC," which packed a Cas9 and sgRNA into EVs using an HIV-derived Gag protein. To recruit the Cas9 RNP into EVs, the authors used a chemical-induced dimerization system named FKBP12 and FRB. To attract the Cas9 protein into the EVs, the Cas9 and Gag proteins were fused to a heterodimerizer and conditionally dimerized by the addition of an inducible chemical ligand. Their results showed that NanoMEDIC achieved more than 90% exon-skipping efficiency in skeletal muscle cells derived from DMD patient iPS cells. Recently, Watanabe et al.^85^ published a protocol for large-scale production of NanoMEDIC, which brought them one step closer to the possibility of *in vivo* applications.

Some teams have encapsulated the unaltered CRISPR-Cas9 protein into purified EVs that were purified from serum by physical and non-virus approaches and further modified EVs with tissue-specific peptides to target the desired organs or tissues as described below.^88^^,^^89^ Moreover, Busatto et al.^43^ incubated Cas9 protein with di-octadecyl-amido-glycyl-spermine, a cationic lipid that binds to protein to form synthetic LNPs, and subsequently incubated with EVs to form a hybrid system to delivery Cas9 protein. The result showed that the Cas9-loaded EVs were efficiently taken up by receipt cells, and 83% of receipt cells were positive for Cas9-Alexa Fluor 488 at a dose of 2 × 10^4^ EV/cell.

There are other studies mentioned that use Cas9 RNP complexes loaded into EVs to measure and track their distribution and uptake in cells. Ye et al.^86^ engineered tumor cells to release EVs containing a Cas9 protein and sgRNA. The RNPs deleted a stop codon before a fluorescent protein, leading to the fluorescence of the reporter cells or reporter animal models. The authors showed that tumor cells and normal cells could communicate with one another through EVs *in vitro*. Moreover, they demonstrated *in vivo* that intravenously injected EVs were absorbed by the liver. They also showed that intravenously injected EVs produced from melanoma xenografts preferentially target the brain and the liver. In addition, Strohmeier et al.^87^ overexpressed CRISPR-Cas9-GFP and GFP-CD63 into 293T cells. Their results demonstrated that there were more single-GFP-labeled EVs (83%) in the EVs separated from CRISPR-Cas9-modified cells than in the EVs derived from GFP-CD63 over-expressing cells (36%). This approach provides a strategy to track tumor derived EVs.

The approaches for helping the RNPs into EVs are summarized in Table 3. Likewise, we also provide some references that review how to modify and deliver proteins into EVs.^80^^,^^90^^,^^91^^,^^92^^,^^93^^,^^94^

**Table 3:** Delivery of CRISPR-Cas9 RNP by EVs

Method	EV source	Target organ/cells	Target gene	Loading method	Reference
Pre-isolation	Expi293F-derived EVs	HEK293T	fluorescent protein	transfection (PEI Max)	Osteikoetxea et al.^76^
	HEK293T-derived EVs	HEK293T-eGFP cells	fluorescent protein	transfection (calcium phosphate)	Whitley et al.^77^
	HEK293T cells derived EVs	U2OS GFP-negative cells	fluorescent protein	Fugene 6 (Promega) and TurboFect	Wang et al.^78^
	HEK293T cells derived EVs	HEK293T-derived HBB-IL2RG EGFP reporter cells	*HBB/IL2RG* and *DMD* exon 53	Fugene HD (Promega)/polyethylenimine	Yao et al.^75^
	293T cells derived Gectosomes	MEF cells	*PINK1 or PCSK9*	transfection (polyethylenimine)	Zhang et al.^135^
	HEK 293T cell-derived exosome	A549stop-DsRed reporter cell line	stop-DsRed	transfection (Lipofectamine 2000)	Ye et al.^136^
	Hepatic stellate cells-derived exosome	Liver	*PUMA*, *CcnE1*,*(KAT5*	electroporation	Wan et al.^137^
	HEK293T cells	Skeletal muscle cells	*DMD*	transfection (Lipofectamine 2000)	Gee et al.^84^
	MSCs-derived exosome	HEK293T-EGFP cells	fluorescent protein	Lipofection (chemical) and electroporation (physical)	Hazrati et al.^138^
	Tumor cells	Liver	fluorescent protein	transfection (Lipofectamine 2000)	Ye et al.^86^
	HEK293T	HeLa cell	fluorescent protein	transfection (Endofectin Max reagent)	Strohmeier et al.^87^
Post-isolation	HEK293T cells-derived	Muscle	*DMD*	transfection (Lipofectamine 2000)	Watanabe et al.^85^
	C2C12 cells	Muscle	miR-29b	transfection (PEI MAX)	Chen et al.^89^
	HEK293T cells-derived	Tumor	*WNT10B*	sonication/freeze-thaw cycles	Zhuang et al.^139^
	Human or mouse serum	Mouse Muscle	*DMD*	protein transfectant	Majeau et al.^88^

### EV surface functionalization

#### Cell type

The origin of an EV is an important factor to consider when planning to use it to deliver gene therapies.^95^ Depending on which cells they originated from, EVs will have different contents, exert different functions, and distribute differently.^96^ First, EVs naturally inherit parent cell features.^95^ Since EVs are frequently used between cells to communicate within the same organ, EVs are more likely to go to the same type of cell from which they originated. EVs also prefer to go to cells that are histogenetically close to them. For example, EVs from microglia tend to reach the CNS, EVs from Schwann cells tend to target the peripheral nerve, and EVs from tumor cells tend to reach homologous tumor cells.^97^^,^^98^^,^^99^ EVs are also used by cells to communicate between cells from different organs or types of cells. For example, EVs derived from mature dendritic cells (DCs) can reach tumor cells.^100^

Wiklander et al.^96^ studied the biodistribution of EVs derived from C2C12 (mouse muscle cell line), B16F10 (melanoma cell line), immature bone marrow-derived DCs, oligodendrocytes from rat (OLN-93), primary human MSCs, and HEK293T in mice following their intravenous injection. C2C12-derived EVs displayed a high liver accumulation (71%) and a low accumulation in the lungs (5%). They were also distributed into the gastrointestinal tract at 8% and into the spleen at 12%. Of B16F10-EVs, 56% were distributed to the liver, 15% to the gastrointestinal tract, 12% to the spleen, and 13% to the lungs. DC-EVs had the lowest liver accumulation at 46%; 28% were distributed into the spleen, 10% into the lungs, and 10% into the gastrointestinal tract. Curiously, EVs from another animal (rat or human) were distributed similarly from the mouse-derived EVs; 65% of the OLN-93-EVs reached the liver and 11% reached the gastrointestinal tract. MSC-EVs were distributed at 71% in the liver and at only 3% in the gastrointestinal tract. HEK293T-EVs accumulated at 60% in the liver and at 16% in the gastrointestinal tract. In their study, Wiklander et al.^96^ showed that DC-EVs have the most prominent distribution to the spleen. Thus, EVs seem to adopt the homing pattern of the parental cell of origin. The fact that EVs may share surface receptors and matrix-binding proteins with their parent cell can explain this natural tropism. For example, immunological cells preferentially target the spleen and other sites exhibiting immunological activity.^101^ Aside from that, another study showed that the epithelial cell-derived micro-vesicles delivered intravenously had demonstrated organ-specific tropism and are known to preferentially aggregate in a few important organs, including the liver, lung, and pancreas.^102^

#### Peptides

Decorating the surface of EVs with proteins or peptides can favor their delivery to specific cells or organs. To engineer the surface of EVs, different strategies can be used. The first strategy can be done before EV isolation. It consists of fusing a transmembrane EV protein to a peptide that can target specific cells. Integrins, lactadherin, lysosome-associated membrane protein-2b, and tetraspanins (CD9, CD63, and CD81 and CD82) are some examples of transmembrane proteins in EVs that can be fused to the desired peptide.^103^ The second strategy to decorate EVs is used after the isolation of the EVs and consists of attaching the peptide to the vesicle surface through adsorption. Copper-catalyzed azide-alkyne cycloaddition (click chemistry)^104^^,^^105^ and cloaking^103^ are some examples of methods that can be used to add peptides to the surface of EVs.

##### Brain targeting

RGE, RVG, T7, ApoB, and ApoA-I mimetic peptides are some examples of peptides that can be added to the surface of EVs to target the CNS (Table 4). Jia et al.^104^ bound RGE peptides (RGERPPR) to the surface of EVs by click chemistry. Their engineered EVs showed a strong glioma-targeting ability.^104^ This was explained by the fact that RGE peptides are specific for the neuropilin-1 receptor which is expressed on glioma. Alvarez-Erviti et al.^106^ decorated EVs with the rabies viral glycoprotein (RVG) peptide. To do so, they cloned it into Lamp2b. Their engineered EVs showed a high tropism for the CNS. Kim et al.^107^ used the T7 peptide which is a type of transferrin receptor (TfR)-binding peptide. The sequence of this peptide is HAIYPRH. The authors conjugated the T7 to Lamp2b for targeting glioblastoma. Their engineered EVs showed a higher targeting of intracranial glioblastoma in rat models than unmodified exosomes or even RVG-labeled exosomes.^107^ Choi et al.^108^ decorated exosomes with ApoB by conjugating it with CD9. The authors observed a higher accumulation of their engineered EVs in the brain compared to control exosomes.^108^ Those EVs even showed prolonged retention in the brain for 24 h.^108^ Ye et al.^109^ used an active targeting strategy by adding ApoA-I mimetic peptides on the surface of EVs. Those peptides can bind to low-density lipoprotein receptors expressed on the glioblastoma cells, and thus target brain cancer cells.

**Table 4:** Some examples of peptides that can be added on EV surface to target specific organs/cells

Targeted cells/organs	Peptide	Reference
Glioma	RGE	Jia et al.^104^
CNS	RVG	Alvarez-Erviti et al.^106^
Intracranial glioblastoma	T7	Kim et al.^107^
Brain	ApoB	Choi et al.^108^
Glioblastoma	ApoA-I mimetic peptide	Ye et al.^109^
Muscles	CP05	Gao et al.^110^
Muscles and heart	T9	Wiklander et al.^46^
Muscles and heart	M12	Nance et al., Gao et al.^140^^,^^141^
Cardiomyocytes	IL-3	Bellavia et al.^115^
Cardiomyocytes	cardiomyocyte-specific peptide	Mentkowski et al.^114^
Heart	CTP	Zahid et al.^116^
Heart	PCM	Nam et al.^117^
Heart	ischemic myocardium-targeting peptide	Zhu et al.^112^
Heart	cardiac homing peptide	Vandergriff et al.^113^
Cartilage	chondrocyte affinity peptide	Liang et al.^51^
Pancreas	tetraspanin-8	Nazarenko et al., Rana et al.^118^^,^^119^
Cancer cells	GE11	Ohno et al.^120^
Cancer cells	TfR	Song et al.^103^
Cancer cells	glycans	Berenguer et al.^122^
Monocyte-derived DCs	MUC1	Koning et al.^123^

##### Muscles targeting

Gao et al.^110^ designed EVs targeting muscles *in vivo*. They fused the CP05 peptide (CRHSQMTVTSRL) with M12 (a muscle-targeting peptide) and conjugated the CP05 peptide with phosphorodiamidate morpholino oligomer (PMO, a treatment for DMD currently approved by the US Food and Drug Administration). The two constructions were co-incubated with exosomes. M12-CP05 and CP05-PMO were able to bind to EVs because of the intrinsic property of CP05 to bind to CD63 on the surface of EVs. Those EVs were injected intravenously into the mdx mouse. Their engineered EVs effectively targeted muscles *in vivo* and even led to a functional improvement in the mdx mice.^110^ The T9 peptide^46^^,^^111^ (SKTFNTHPQSTP) can also be used to target skeletal muscles and the heart.

##### Heart targeting

Zhu et al.^112^ put an ischemic myocardium-targeting peptide (CSTSMLKAC) on EVs to preferentially target ischemic injured cardiomyocytes. Vandergriff et al.^113^ decorated EVs with the cardiac homing peptide (CSTSMLKAC). The authors improved the efficacy of delivery using their engineered EVs. Mentkowski et al.^114^ fused a cardiomyocyte-specific peptide (WLSEAGPVVTVRALRGTGSW) to Lamp2b. Their engineered EVs showed an increased cardiomyocyte uptake. Interleukin-3 can be fused to Lamp2b in EVs to target cardiomyocytes.^115^ To target the heart, EVs could be decorated with the CTP peptide^116^ and the PCM peptide.^117^

##### Cartilage targeting

Liang et al.^51^ fused a CAP with Lamp2b to generate engineered EVs for cartilage arthritis. By intra-articular injection of their engineered EVs containing the plasmid of Cas9 and sgRNA target to MMP-13, they successfully targeted the deep region of the cartilage matrix in arthritic rats and knocked down the *MMP-13* in chondrocytes. It resulted in the ablation of MMP-13 expression and in the attenuation of the hydrolytic degradation of the extracellular matrix proteins in the cartilage.

##### Pancreas targeting

In the pancreas, CD54^+^ acts as a key ligand that could attract the EVs modified with tetraspanin-8 on the surface, a multimodular protein involved in clathrin-mediated. Its specificity can be explained by its capacity to form a complex with the integrin α4 or CD49d.^118^^,^^119^

#### Tumor cell targeting

Tumor cells express specific peptides on their surface. By finding peptides that bind specifically to those molecules and by adding them to the EVs’ surface, the resulting EVs could target cancer cells. For example, the epidermal growth factor receptor (EGFR) is highly expressed on the surface of tumor cells. The GE11 peptide can bind specifically to EGFR. Thus, the GE11 peptide can be added to the EV surface to target cancer cells.^120^

Zuo et al.^121^ used exosomes derived from DCs and decorated them with an HCC-targeting peptide (P47-P), an α-fetoprotein epitope and a functional domain of high-mobility group nucleosome-binding protein 1 (N1ND-N). N1ND-N can be used to recruit and activate DCs.

EVs can also be decorated with glycans.^122^ Cancer cells have the chemokine (C–C motif) receptor 8, and the soluble ligand chemokine (C–C motif) ligand 18 on their surface, and glycans can bind to those proteins.^122^ Another protein that is present in cancer cells is Tf. TfRs can be found naturally on the surface of EVs.^103^ Thus, we can hypothesize that non-engineered EVs can naturally target cancer cells.

##### Monocyte targeting

Monocyte-derived DCs present intercellular adhesion molecule-grabbing non-integrin on their surface. This protein can bind to soluble mucin 1 (MUC1). Thus, EVs can be decorated with peptides from MUC1 to target monocyte-derived DCs.^123^

### Antibody-based surface functionalization

Antibody-based peptides are another type of peptide that has been used to increase the targeting ability of EVs, especially when targeting cancer cells. For example, Cheng et al.^124^ developed synthetic multivalent antibody-targeted exosomes. These EVs expressed the monoclonal antibody αCD3 UCHT1, which is specific for CD3 T cells, and single-chain variable fragments of αEGFR cetuximab, which is specific for cancer cell-associated EGFR. The authors demonstrated that these engineered EVs could efficiently target T cells and EGFR-expressing breast cancer cells.

### Aptamer

Another way to make EV delivery more accurate is by using aptamers. Aptamers bind to specific ligands with high affinity.^103^ For example, aptamers have been used to target cancer cells and even the brain. Han et al.^125^ decorated EVs with E3 aptamers and succeeded in targeting prostate cancer cells *in vitro* as well as *in vivo*. Xiang et al.^126^ added epithelial cell adhesion molecule aptamers on the surface of EVs and succeeded in penetrating tumors effectively. Macdonald et al.^127^ used a dual-functional aptamer combining EpA and TfR. Their EVs successfully passed the blood-brain barrier (and delivered the drug to the brain. Ren et al.^128^ used aptamer targeting α-synuclein aggregates to target the brain.

### Increasing EV encapsulation and delivery efficiency

#### Increasing circulation time

To decrease phagocytosis by immune cells, CD47, an integrin protein, can be added to the surface of EVs.^103^ This also improves the stability of the EVs in the circulatory system. CD47 allows the release of a "do not eat me" signal by binding to signal regulatory protein-α on the macrophage surface.^129^ Polyethylene glycol (PEG) is also known to allow an increased circulation time when added to a particle.^130^ It was shown that PEGylation decreases nonspecific interactions with cells and increases EV circulation time.^131^

#### Autophagy pathway inhibitors

The lysosomal degradation of EVs can decrease the efficiency of EV delivery. In lysosomes, the cargo of EVs might be enzymatically degraded.^132^ This phenomenon happens mostly through the autophagy pathway.^133^ To overcome this obstacle, Zhang et al.^134^ inhibited the autophagy pathway. With this strategy, they improved the drug delivery of EVs in recipient cells. They studied the delivery of plasmid DNA and proteins, and the delivery of the two forms was enhanced by using an inhibitor of the autophagy pathway.^134^

### Conclusion and perspective

Efficient *in vivo* CRISPR-Cas9 delivery is a significant challenge. Fortunately, there has been a notable advancement in the use of EVs for CRISPR-Cas9 delivery in the past few years. EV-based delivery approaches have several advantages over traditional virus delivery systems. For instance, EVs have greater stability in blood, enabling long distance transmission. EVs are often specific to cells or tissues and can get through a variety of physiological barriers and cellular obstacles. EVs can even be further functionalized to provide improved targeting capability. Given these benefits, EVs are regarded as a powerful method of delivering therapeutic substances *in vivo*.

However, there remain many technological obstacles to overcome before EV-mediated gene therapies may be used in a clinical setting. Even though EVs can be functionalized to provide targeting capability, the EVs injected by intravenous injection generally go to the liver. Thus, further studies to improve cell and tissue targeting must be completed. In addition, the various modifications that can load cargo into EVs, most of these have not yet been used to load CRISPR-Cas9. Currently, there are no clinical trials using EVs that deliver CRISPR-Cas9 for treatments. To increase the effectiveness of gene editing *in vivo*, higher efficiency loading methods specific to encapsulating CRISPR-Cas9 system into EVs are required. Solutions to increase EVs uptake, cargo release into the cytoplasm, and endosomal escape must be found to improve gene editing efficacy in a therapeutic context. Together, EVs are promising vectors for delivering the CRISPR-Cas9 system *in vivo*, but multiple technical hurdles await.
